# The role of substance P in epilepsy and seizure disorders

**DOI:** 10.18632/oncotarget.20606

**Published:** 2017-09-01

**Authors:** Xue Feng Wang, Tong Tong Ge, Jie Fan, Wei Yang, Ran Ji Cui

**Affiliations:** ^1^ Jilin Provincial Key Laboratory on Molecular and Chemical Genetic, Second Hospital of Jilin University, Changchun, People's Republic of China

**Keywords:** substance P, epilepsy, inflammation, neuron

## Abstract

A range of evidence implicates the neuropeptide substance P (SP), a member of the tachykinin family, in emotional behavior, anxiety, pain, and inflammation. Recently, SP has been implicated in susceptibility to seizures, for which a potential proconvulsant role was indicated. Indeed, antagonists of a specific SP receptor, neurokinin-1 receptor, were found to attenuate kainic acid (KA)-induced seizure activity. However, detailed mechanisms of SP regulation in epilepsy remain obscure. In this review, we summarize the present literature to expound the role of SP in epilepsy, and provide hypotheses for potential mechanisms.

## INTRODUCTION

Epilepsy is a common chronic neurological disease characterized by the occurrence of recurrent and unprovoked seizures [1]. Of the more than 70 million people around the world suffering from diverse types of epilepsy [2], approximately 30% are resistant to therapy with currently available traditional antiepileptic drugs (AEDs) and, thus, still have frequent seizures [3]. As such, novel and effective therapeutic targets for the development of innovative AEDs are needed.

Hyperexcitability resulting from disruption of the balance between excitation and inhibition in nervous tissue can trigger acute seizures [4]. Imbalances cause excessive synchrony of electrical discharges from specific neurons, thus triggering a series of abnormal behaviors [5]. Inhibitory/excitatory transmission systems play a major role in such imbalances, especially the inhibitory neurotransmitter gamma-aminobutyric acid (GABA) and the excitatory neurotransmitter glutamate [6, 7]. Most traditional AEDs target ion channels or ionotropic receptors to decrease the excitatory tone or increase the inhibitory tone to correct imbalances [8, 9]. However, wide distribution of neurotransmitter receptors throughout the human brain allows traditional AEDs to influence potentially numerous other normal neurons while exerting their antiepileptic actions [10].

Compared with common AEDs, targeting neuropeptide receptors yields higher selectivity because these receptors exhibit more refined localization, which can also help avoid harmful side effects [11]. Recently, numerous neuropeptides were confirmed to affect the pathogenesis of epilepsy including ghrelin, galanin, and neuropeptide Y, which were found to suppress seizures [12–15]. Other neuropeptides, such as corticotropin-releasing hormone, encephalin, and tachykinins, have demonstrated proconvulsant properties [16–18]. Substance P (SP), a member of the tachykinin family, is well known to facilitate epileptic activity in experimental animal models of epilepsy via activation of specific receptors [19]. Intrahippocampal SP injection was reported to profoundly enhance susceptibility to seizures and trigger self-sustaining status epilepticus (SSSE), indicating a potential proconvulsant effect of SP [20]. In addition, antagonists of specific SP receptors were found to attenuate KA-induced seizure activity [21]. Therefore, specific SP receptor antagonists are novel, practical, and potentially valuable targets for epilepsy treatment.

However, fundamental molecular and cellular mechanisms underlying the proconvulsant role of SP remain obscure, necessitating further experimental and clinical research. In this review, we summarize present literature describing regulation of the neuropeptide SP in epilepsy, as well as therapeutic properties of specific receptor antagonists, to provide perspective for novel AED development.

### SP and neurokinin-1 receptor

SP is an undecapeptide member of the tachykinin neuropeptide family [22]. Tachykinins, which share a specific C-terminal sequence, are one of the largest neuropeptide families. In addition to SP, neurokinin A (NKA) and neurokinin B (NKB) are closely related neuropeptides. Euler and Gaddum first isolated SP from horse brain and intestine in 1931, when they also reported hypotensive and contractile effects on gastrointestinal smooth muscle [23, 24]. Chang and Leeman subsequently elucidated the amino acid structure of SP [25]. SP is produced from the preprotachykinin-A (*PPT-A*) gene, which also encodes NKA [26]; whereas, the preprotachykinin-B (*PPT-B*) gene solely encodes NKB [27]. The *PPT-A* gene is spliced into four different mRNAs (α, β, γ, and δ) via alternative transcription. All four isoforms can encode SP, while γ-PPT and δ-PPT only encode NKA [28]. SP is widely distributed throughout the central nervous system (CNS), including the brain and spinal cord [29]. In the CNS, SP immunoreactivity has been observed within the rhinencephalon, telencephalon, basal ganglia, hippocampus, amygdala, septal areas, diencephalon, hypothalamus, and mesencephalon [22, 30, 31]. Importantly, the hippocampus and cortex are well known to be epilepsy-prone brain regions.

Tachykinins exert numerous neurophysiological functions, primarily by binding to neurokinin (NK) receptors including neurokinin-1 receptor (NK-1R), NK-2R, and NK-3R. All three receptors are G protein-coupled receptors with seven transmembrane domains. The endogenous receptor for SP is NK-1R, as SP selectively binds to NK-1R with higher affinity than NK-2R and NK-3R [25, 27, 32]. In the CNS, NK-1R is mainly expressed in the caudate-putamen, superior colliculus, and nucleus accumbens, with moderate to low levels of NK-1R found in the inferior colliculus, olfactory bulb, hypothalamus, hippocampus, substantia nigra, and cerebral cortex [33]. Interestingly, an apparent mismatch exists between CNS distribution of SP and NK receptors [34, 35]. SP is highly expressed in the substantia nigra, where NK-1R is rarely detected [36]; although, technical limitations might explain this mismatch. Upon binding to NK-1R, SP can cause a rapid internalization action, whereby the receptor–ligand complex translocates from the plasma membrane into the cytoplasm [37]. This internalization process is reversible with complete return of internalized receptors to the surface [38]. Notably, as a neuropeptide, SP can be transported to activate distant target neurons after secretion despite the low expression of SP and NK-1R in seizure-prone regions.

### SP and epilepsy

Recently accumulating evidence implicates SP in the facilitation of epileptic activity in various experimental models of epilepsy [39]. As summarized in Table 1, release and expression of SP was increased or reduced following epileptic episodes. One clinical study revealed elevated SP levels in the serum and cerebrospinal fluid of children with seizure disorders [40]. Other researchers observed a reduction of SP-like immunoreactivity in epileptic animal models after acute seizure induction, which subsequently reversed to normal levels over time. The tendency for hyperstimulated neurons to absorb available neuropeptides for efficacious neuroprotection might be an explanation. In addition, intrahippocampal administration of SP triggered SSSE under subthreshold stimulation, indicating increased susceptibility to epilepsy [20]. Liu H et al. observed significant reductions of seizure duration and severity induced by KA/ pentylenetetrazol in *PPT-A*–deficient mice. KA is known to directly stimulate glutamate receptors and cause hippocampal neuronal loss during epileptogenesis. *PPT-A* deficiency can decrease KA-induced hippocampal damage and downregulate Bcl-2 associated X protein (Bax) and caspase protein expression, indicating potential involvement of the SP gene in regulation of neuronal damage in epileptogenesis [41]. In addition, intrahippocampal injection of SP alone or extracts of cysticercosis granuloma (a helminth brain infection known to cause seizures [42]) obtained from infected wild-type mice induced fatal seizures in mice; whereas, mice injected with extracts from infected SP precursor-deficient mice survived induced seizure activity [43].

**Table 1:** Overview of SP release or expression following epilepsy

Models	Schedule	Main findings	Ref
**KA (rats)**	SP-like immunoreactivity 3 h after KA injection	SP-like immunoreactivity ↓ (40%–50%) in frontal cortex and hippocampus	[49]
	SP-like immunoreactivities 10–30 days after KA injection	SP-like immunoreactivity ↑ in striatum and substantia nigra	
**KA (rats)**	SP-like immunoreactivity 3 h after KA injection	Total neurokinin (A + B) and SP immunoreactivity ↓ (25%–40%) in frontal cortex, dorsal hippocampus, and striatum	[50]
	SP-like immunoreactivity 30 days after KA injection	SP levels ↑ (30%) in frontal cortex	
**Human**	70 patients aged from 1 month to 18 years with seizure disorders	Serum SP levels ↑	[40]
**Pilocarpine (mice)**	Pilocarpine administrated (i.p.) to 2/3/4/9-week-old rats to induce SE	In CA1, SE-induced SP increment is age-dependent, maximal expression occurred in 2-week-old rats and progressively decreased in 3- and 4-week-old rats, and adults; In CA3 and dentate granule cell layer, SE induced minimal increases in SP in 2-week-old rats	[51]
	*PPT-A* mRNA 2 h after injection	*PPT-A* mRNA ↑ in granule cells, CA3 and CA1 pyramidal cell layers of hippocampus	
**Perforant path stimulation(rats)**		SSSE induced novel expression of SP-like immunoreactivity in hippocampal principal cells	[52]

KA, kainic acid; I.P., intraperitoneal injection; SE, status epilepticus; SSSE, self-sustaining status epilepticus; ↑, increased; ↓, decreased.

Electron microscopic analyses revealed the synaptic input of SP receptor-positive dendrites to be increased in the epileptic CA1 region, while ratios of inhibitory and excitatory synaptic inputs were unchanged [44]. In literature analyzing surgically removed hippocampi of patients with temporal lobe epilepsy (TLE), SP receptor-immunoreactive cells were mainly preserved in the non-sclerotic CA1 region, while their number was decreased in sclerotic tissue [45]. Additionally, altered morphology of SP receptor-immunoreactive cells was observed, including more dendritic branches. Increasing amounts of recent research support the contribution of structural changes in hippocampal synaptic plasticity to the development of epilepsy. Mossy fiber sprouting, a general phenomenon, is regarded as a symbol of lesions indicating secondary epilepsy [46, 47]. Therefore, SP may worsen epileptic activity by modulating hippocampal dendritic inhibition/excitation and axonal sprouting. However, distinct molecular mechanisms have yet to be elucidated.

Recent literature has described increased NK-1R expression in both the ipsilateral and contralateral hemispheres of patients with TLE. Indeed, a positive correlation between NK-1R expression and seizure frequency was reported in the medial temporal lobe [48]. Collectively, numerous studies have shown that the neuropeptide SP can facilitate epileptic activity via SP/NK-1R signaling. Although, as evidence describing molecular mechanisms is lacking, more investigation is needed.

### Possible mechanisms of SP/NK-1R signaling in epilepsy

### SP and glutamate-induced excitotoxicity

Glutamate-induced excitotoxicity plays a significant role in epileptogenesis [53]. Excitotoxicity is the pathological process by which neurons are damaged or killed by excessive and sustained stimulation by excitatory neurotransmitters, primarily glutamate and similar substances [53–56]. Glutamate-induced excitotoxicity may reinforce imbalances between the inhibition and excitation of neurotransmission and, thus, indirectly potentiate epilepsy [57].

Several studies have reported the involvement of SP in neuronal sensitization through activation of NK-1R in postsynaptic dorsal horn neurons. SP was reproted to potentiate N-methyl-D-aspartate (NMDA)-induced transient Ca^2+^ currents recorded in acutely isolated neurons from rat dorsal horn [58]. Early research revealed that SP depolarizes the membrane by reducing inward rectifying K^+^ currents [59]. More recent literature has revealed that this potentiation results from an increased magnitude and prolonged duration of NMDA-responsive neurons, rather than an increased percentage of NMDA-responsive neurons [60]. Wu and colleagues demonstrated that sustained potentiation of NMDA receptors by SP was time-dependent and could be blocked by extracellular application of a selective non-peptide NK-1R antagonist (WIN51708) [61]. SP-induced intracellular Ca^2+^ currents can increase glutamate sensitivity by promoting protein kinase C (PKC)-dependent phosphorylation of NMDA receptors [62]. SP selectively suppressed GABA(A) receptor-mediated inhibitory post-synaptic currents in striatal cholinergic interneurons and the NK-1R antagonist RP67580 attenuated this suppression [63]. SP has also been reported to prolong excitatory postsynaptic potentials (EPSPs) by further depolarizing the membrane of spinal cord neurons [64].

Promotion of glutamate release by SP has been widely reported in several brain regions, including the suprachiasmatic nucleus [65], striatum [66], and nucleus tractus solitaries [67]. Glutamate also exerts positive feedback on SP release by activating NMDA receptors [68]. Thus, SP can increase the potency and efficacy of glutamatergic synaptic transmission to indirectly trigger seizures. As seizures can increase extracellular glutamate and subsequent excitotoxic damage, this reinforces the occurrence of more seizures [69]. Unfortunately, as little evidence of an interaction between SP and glutamate receptors has been reported in epilepsy-related brain regions, further research is needed to determine the relationship between SP and glutamate-induced excitotoxicity.

### *PPT-A* gene and epileptogenesis

As reviewed above, *PPT-A* deficiency can significantly reduce the duration of seizures and the expression of Bax and caspase proteins in KA-injected mice [41]. *PPT-A* mRNA expression was dramatically promoted in principal neurons of CA3, CA1, and the dentate gyrus (DG), as well as in hippocampal mossy fibers during SSSE [20, 51]. Wasterlain et al. observed similar results in *PPT-A* knockout mice, which displayed seizures of much shorter duration and lesser intensity after KA injection [52]. *PPT-A* encodes both the precursor of SP and NKA. As NKA and its receptors are sparsely expressed in the brain, it is likely that SP exerts the bulk of neurophysiological actions [70]. Subsequent research demonstrated that KA injections result in transient upregulation of *PPT-A* mRNA within 3 hours after injection [71], indicating the *PPT-A* gene might be a potential proconvulsant gene. Repressor element-1 silencing transcription factor, a candidate modulator of gene expression during status epilepticus, altered expression the *PPT-A* gene and may, therefore, regulate the progression of epileptogenesis in KA-injected rats [71].

Recently, there has been increased recognition of the genetic components participating in the etiology of generalized/focal epilepsies [72]. However, a large number of susceptibility genes for genetic epilepsies remain unknown [73]. The proconvulsant effects of SP have been extensively studied. The *PPT-A* gene, which encodes SP, exhibits a rapid and transient response to KA-induced excitation, which might contribute to the formation of excitable circuits in the latent period of epileptogenesis. However, as studies focusing on deep mechanisms of the relationship between *PPT-A* and epilepsy are lacking, more investigation is needed to better understand the role of *PPT-A* in epileptogenesis.

### SP and neurogenic inflammation

Growing evidence indicates that inflammation and acute CNS injury promote epileptogenesis [74]. Indeed, a clear correlation between the severity of injury and likelihood of epilepsy has been described [75]. Head trauma, stroke, tumors, and febrile seizures during childhood can lead to the onset and development of epilepsy [76]. Such initial injuries often lead to subsequent secondary brain injury by triggering inflammation [77]. Histopathological changes occurring after initial precipitating events, including neuroinflammation, vascular damage, blood-brain barrier (BBB) leakage and leukocyte infiltration, contribute to epileptogenesis [78, 79]. The proinflammatory role of SP has been widely reported [80]. SP can influence a wide range of inflammatory diseases in respiratory, gastrointestinal, and musculoskeletal systems [80]. In the CNS, consistent with observed increases after acute seizures, SP immunoreactivity was found to be stimulated both perivascularly and in cortical neurons and astrocytes following diffuse traumatic brain injury in both animal and clinical studies [81, 82]. Elevated SP can directly increase BBB permeability and subsequent plasma protein extravasation [81]. Moreover, SP can promote the expression of adhesion molecules, allowing an increased number of inflammatory cells to cross the BBB into the brain parenchyma [28]. In addition, SP can activate microglia and astrocytes to contribute to increased BBB permeability [80, 82].

Although increasing amounts of research have focused on the neurogenic inflammatory effects of chronic epilepsy, the underlying relationship is still debatable. What has been determined is that both primary injury and subsequent inflammation can lead to secondary injuries exhibiting positive feedback with epilepsy. SP can promote inflammation and indirectly worsen the effect of injury on epilepsy. Fortunately, the proinflammatory actions of SP can be blocked by NK-1R antagonists, which are thought to be a promising therapeutic for neuroprotection.

### NK-1R is a promising therapeutic target for treating epilepsy

As reviewed above, activation of the SP/NK-1 signaling pathway contributes to the pathophysiology of epilepsy. Several studies have revealed that NK-1R antagonists are efficacious for inhibiting epileptic activity. NK-1R antagonists can bind to NK-1R to block the pathophysiological effects of SP [83].

NK-1R was extensively studied as a potent neuropeptide receptor for therapeutics [84]. Although selective NK-1R agonists have been developed, few have been found to have therapeutic potential, unlike NK-1R antagonists [85]. Two types of NK-1R antagonists can be classified according to their chemical composition, including peptide and non-peptide NK-1R antagonists [70]. The first binds to NK-1R located on the extracellular ends of transmembrane helices [86], while the latter binds to deeper regions between the transmembrane segments [87]. Aprepitant and fosaprepitant are the only NK-1R antagonists currently available in clinical practice, where they are prescribed as antiemetics [88].

Published studies identifying the antiepileptic effects of NK-1R antagonists in various animal models are summarized in Table 2. Garant and colleagues first reported convulsions to be alleviated by intranigral microinfusion of NK-1R antagonists in electroshock/intravenous bicuculline-treated rats [89]. Similarly, the NK-1R antagonist CP-122,721-1 was found to decrease seizure activity in KA-kindled rats. In addition, this antagonist also inhibited KA-induced neuron death within the hippocampal CA1 subregion [21]. A recent study reported that aprepitant can decrease neuronal cell death and oxidative stress in bilateral common carotid artery-occluded rats [90]. Interestingly, GR205171 (vofopitant), a NK-1R antagonist, demonstrated potent antiepileptic effects as a Na^+^ channel blocker, rather than exerting direct antiepileptic efficacy [91]. Thus, NK-1R antagonists may elicit synergistic effects with other traditional AEDs, which could be a promising target for treatment of refractory epilepsy. Collectively, these results indicate that possible mechanisms of antiepileptic effects may be related to the potential neuroprotective efficacy of NK-1R antagonists, although cellular and molecular mechanisms remain unknown. Despite beneficial effects of NK-1R antagonists being widely reported in animal studies, there is a lack of human studies. Thus, specific mechanisms underlying the antiepileptic efficacy NK-1R antagonists require further investigation.

**Table 2:** Overview of NK-1R antagonist effects in various experimental models

Antagonist	Type	Model	Main findings	Ref
**SP antagonists**		Electroshock/ intravenous bicuculline (rats)	Significantly attenuated convulsions	[89]
**CP-122,721-1**	Non-peptide	KA (i.p. rats)	Possibly decreased seizure activity; Counteracted KA-induced nerve cell death in CA1	[21]
**Spantide II and RP-67,580**		Bipolar electrode stimulation in DG (rats)	Reduced seizure duration in a dose-dependent manner; Suppressed EEG frequency	[20]
**GR205171 (vofopitant)**		Electroshock (rats)	Exhibited no anticonvulsant efficacy by itself; Potentiated the anticonvulsant efficacy of lamotrigine and other sodium channel blockers	[91]

KA, kainic acid; EEG, electroencephalographic; DG, dentate gyrus.

Compared with traditional AEDs, neuropeptide receptor antagonists are safer and elicit fewer side effects [92]. Thus, future investigations should pay more attention to novel NK-1R antagonists, which are remarkably stable and can transport freely across the BBB.

## CONCLUSIONS

The neuropeptide SP reportedly participates in many pathophysiological processes, such as emotional behavior, depression, anxiety, vomiting, pain, inflammation, and cancer. Several studies have implicated the neuropeptide SP in epilepsy. In this article, we reviewed the present literature about regulation of SP in epilepsy. Mechanisms underlying the proconvulsant role of SP are potentially explained by various aspects. First, SP can potentiate both EPSPs (by further membrane depolarization) and the release of excitatory glutamate, which amplify glutamate-mediated excitotoxicity in epilepsy-suspected brain regions. Second, the *PPT-A* gene, which encodes SP, exhibits a rapid and transient increase in response to KA-induced excitation. Moreover, *PPT-A* deficiency can profoundly reduce the severity of KA-induced acute seizures, potentially implicating the *PPT-A* gene in the generation of epilepsy. Third, SP displays proinflammatory effects in the PNS and CNS. In response to initial brain injury, SP activates microglia and astrocytes, and subsequently decreases BBB permeability to increase inflammation. Despite the fact that the proconvulsant role of SP might not be a direct pathogenetic factor for epilepsy, SP leads to secondary injuries and deterioration that can increase seizure frequency. In addition, as several NK-1R antagonists have been reported to decrease seizure activity in KA-kindled rats, NK-1R may be a promising therapeutic target for treating epilepsy. In conclusion, while the focus of future research is identification of safe and efficient artificial ligands for clinical application, underlying molecular mechanisms of SP regulation in epilepsy require further study.
