# Correction: The role of substance P in epilepsy and seizure disorders

**DOI:** 10.18632/oncotarget.26991

**Published:** 2019-05-28

**Authors:** Xue Feng Wang, Tong Tong Ge, Jie Fan, Wei Yang, Bingjin Li, Ran Ji Cui

**Affiliations:** ^1^ Jilin Provincial Key Laboratory on Molecular and Chemical Genetic, Second Hospital of Jilin University, Changchun, People's Republic of China

**This article has been corrected:** The new Correspondence details are given below:

**Bingjin Li^1^**

***Correspondence to:*** Bingjin Li, **email:** libingjin@jlu.edu.cn

Original article: Oncotarget. 2017; 8:78225–78233. 78225-78233 . https://doi.org/10.18632/oncotarget.20606

